# Fluorescent multiple staining and CASA system to assess boar sperm viability and membranes integrity in short and long-term extenders

**Published:** 2013-03-05

**Authors:** A. Lange-Consiglio, A. Meucci, F. Cremonesi

**Affiliations:** Università degli Studi di Milano, Faculty of Veterinary Medicine, Department of Health, Animal Science and Food Safety, Large Animal Hospital, Reproduction Unit, 6 Via dell’Università, I-26900 Lodi, Italy

**Keywords:** Boar, CASA, Fluorescent staining, Motility, Semen storage

## Abstract

The aim of this study was to assess the effect on boar spermatozoa quality of *in vitro* storage in short and long-term extenders by fluorescent multiple staining (FMS) and computer assisted semen analyzer (CASA). Fresh ejaculates from three healthy, sexually mature boars were diluted with equal volumes of six short-term or three long-term commercial extenders and stored at 19°C for 6 days (short-term) or 12 days (long-term). The integrity of spermatozoa membranes was analyzed by FMS using propidium iodide, 5,5’,6,6’-tetrachloro-1,1’,3,3’ tetraethylbenzimidazolyl-carbocyanine iodide (JC-1) and fluorescein isothiocyanate-conjugated peanut agglutinin (PNA). The results obtained from this staining were compared with spermatozoa motility assessed by CASA. Our study showed that the number of viable spermatozoa with non-reacted acrosomes and intact mitochondria was positively correlated with the rate of motile spermatozoa (r^2^>0.9) irrespective of the extender used. In all extenders the number of motile spermatozoa significantly decreased as preservation period increased (P<0.05). FMS test is a potent indicator of sperm motility because it analyses mitochondrial integrity independently from observable alterations in motility. The best performing extenders were BTS for short-term storage and TRI-x-Cell for long-term storage.

## Introduction

Boar spermatozoa are sensitive to low temperatures, because of their plasma membrane composition (White, 1993), and therefore must be stored at moderately reduced temperatures (*i.e*. 16–20°C). Boar spermatozoa preserved in a liquid medium show morpho-functional changes that resemble the natural ageing process and the severity of these changes is influenced by the conditions and duration of storage. These changes can involve plasma membrane, mitochondria, acrosome membrane and DNA. Therefore, it is important to evaluate whether differences exist between extenders with regard to their capacity to delay such changes. Standard semen analysis uses light microscopy to assess sperm motility and although this analysis is cheap and rapid, the accuracy of the outcome depends on subjective estimations made by individuals (Quintero-Moreno *et al.*, 2004; Vyt *et al.*, 2004).

The CASA are a useful tool for determining the motion characteristics of sperm samples (Quintero-Moreno *et al.*, 2004) because they are objective, independent of the interpretation of the technician and give detailed information on sperm movement. However, more functional analyses are required to make the motility a potent sperm parameter (Love *et al.*, 2003; Vyt *et al.*, 2004). The mitochondrial membrane potential is a sensitive indicator of the energetic state of the mitochondria and the cell, and can be used to assess the activity of the mitochondrial respiratory chain, electrogenic transport systems and the activation of the mitochondrial permeability transition (Ly *et al.*, 2003).

The JC-1 is considered as a fluorochrome which can measure membrane potential with great accuracy in intact cells (Salvioli *et al.*, 1997) including spermatozoa (Troiano *et al.*, 1998). This staining could be a potent indicator of sperm motility because it analyses mitochondrial integrity independently from an eventual temporary manifestation of immotility. This would explain why spermatozoa with intact mitochondria are potentially motile in spite of their transient immotility (Bussalleu *et al.*, 2005). Indeed, Vyt *et al*. (2004) found low LIN (35%) in boars being used successfully in A.I. and Juonala *et al*. (1999) reported results of full fertility in semen samples from Landrace and Yorkshire boars in which the spermatozoa were totally immotile at examination.

In addition to the analysis of motility, other analyses have been designed to study simultaneously the status of the nucleus, the acrosome, and mitochondria of spermatozoa (Cooper and Yeung, 1998; Huo *et al.*, 2002; Huszar *et al.*, 2003). The best results were obtained from combining different specific staining for each sperm compartment (Juonala *et al.*, 1999; Langlois *et al.*, 2005).

The integrity of the plasma membrane is one of the most important aspects of sperm biology, since the plasma membrane is involved in metabolic exchanges with the surrounding medium and also plays an important role in several events that take place during fertilization, such as capacitation, acrosome reaction, hypermotility, and sperm fusion with the oocyte (Cooper and Yeung, 1998; Ducci *et al.*, 2002). Moreover, the integrity of the acrosome membrane is also important because only spermatozoa with intact acrosomes can penetrate the barriers around the ovum.

For these reasons, recently developed techniques for functional analysis of sperm motility focus on assessing mitochondrial activity (Ruiz-Pesini *et al.*, 2000; Love *et al.*, 2003) in addition to the study of integrity of plasma and acrosome membranes. The objective of this study was to investigate the effects of different short and long-term extenders on sperm motility, viability, acrosome integrity and mitochondrial sheath of boar spermatozoa.

## Materials and Methods

Procedures involving animals were performed in accordance with the recommendation of the Bioethics Committee of Milan University.

### Reagents and extenders

All reagents were purchased from Sigma Aldrich (Milan, Italy) except JC-1 purchased from Molecular Probe, Invitrogen (Life Technologies Italia, Monza, Italy).

The extenders used in this experiment were commercial products, so their exact chemical composition is unknown. However, Johnson *et al*. (2000) reported the following recipes for Beltsville Thawing Solution (BTS): 37.0 g glucose, 1.25 g EDTA, 6.0 g sodium citrate, 1.25 g sodium bicarbonate and 0.75 g potassium chloride for 1 L of solution.

Six short-term (MR-A 3, BTS, Androstar-Plus, Modena 1, Mr Pig Sire and ZM) and three long-term (HP2000, Sus and Tri X-Cell) extenders were used and diluted using ultrapure water.

The list of extenders is showed in Table 1.

**Table 1:** Features of the extenders used in this study.

Trade name	Distributor	Fraction	Duration of storage
MR-A 3	Kubus, s.a. and general farm	1	Short term
BTS	Minitub	1	Short term
Androstar-Plus	Minitub	1	Short term
Modena 1	Semenitaly	2	Short term
Mr Pig Sire	Pig service	2	Short term
ZM	Microgene	2	Short term
HP2000	Microgene	2	Long term
Sus	Medichimica	1	Long term
Tri X-Cell	Imv technologies	1	Long term

The pH of each extender and osmolality were measured.

### Semen collection and preparation

Sperm-rich fractions of semen were collected, using the gloved-hand manual collection method, from three boars (Large White), aged between 2 and 4 years that were routinely used in a local AI centre. Boars were housed in individual pens in environmentally controlled buildings. They were given *ad libitum* access to water and were fed commercial diets according to the nutritional requirements for adult boars. Semen from each boar was collected once weekly for three consecutively weeks during the spring season.

The semen samples were transported to the laboratory at 37°C within 30 min in insulated thermos and filtered through four layers of sterile gauze into a pre-warmed beaker to remove gel particles. The fresh semen was evaluated for macroscopic (volume, pH) and microscopic properties (concentration, motility, primary and secondary abnormalities).

The filtered semen of three boars was pooled and divided into equal volumes in three long-term and six short-term extenders in sterile test-tubes (50 mL). Semen was diluted to a concentration of 50 × 10^6^ spermatozoa/mL. After maintenance at room temperature (22°C) for 1 h, the diluted semen was stored with gentle agitation twice daily at 19°C for up to 6 (short-term) or 12 days (long-term) and different end points were assessed every two days. Separate tubes were filled for colony forming unit (CFU) counting prior to any investigation.

Each sample of fresh semen was evaluated through CASA for examination of sperm motility and concentration, then, sperm motility was assessed in pooled diluted samples stored at 19°C at days 0, 2, 4, 6, 8, 10 and 12 of storage. At the same time, the samples were evaluated by FMS using propide iodide (PI), 5,5’,6,6’-tetrachloro-1,1’,3,3’tetraethyl-benzimidazolyl-carbocyanine iodide (JC-1) and peanut agglutinin lectin (FITC-PNA).

### Sperm morphology

Rose bengal/blue Victory staining was used to evaluate sperm morphology in each fresh sample of semen. The semen was smeared on slides pre-heated to 37.5°C. The smears were air-dried and fixed in 10% formalin, rinsed in distilled water, stained for 10 minutes in 3% aqueous solution of rose bengal, rinsed in distilled water, stained for 50 sec in 3% aqueous solution blue Victory, rinsed in distilled water and air dried. The evaluation of sperm was performed with a microscope using a 100X objective.

### Semen analysis using CASA

Motility patterns were assessed in fresh semen and in aliquots of pooled stored semen using the CASA system. A customized CASA system was assembled with microscope fitted with warming stage, negative phase contrast optics (20x objective and 10x ocular for 200x total magnification), and video camera interfaced with a computer to digitize and analyze the images (Lange-Consiglio *et al.*, 2010). The software used for image acquisition and analysis was Image-Pro Plus 5.1-Media Cybernetics (Immagini & Computer, Bareggio, Italy).

Various parameters relating to sperm cell tracks were analyzed using digital image analysis. Before CASA analysis the samples were diluted with the respective extender to approximately 20 × 10^6^ spermatozoa/mL. Sperm trajectories cannot be measured if the spermatozoa concentration is too high because of the high incidence of multiple collisions between cell paths.

The temperature of the sample also influences sperm motility and consequently, CASA-analysis (Iguer-ouada and Verstegen, 2001) since spermatozoa move more slowly at lower temperature. The optimal temperature for analysis of sperm motion is body temperature (37-38°C) (Verstegen *et al.*, 2002), therefore, an aliquot of 5µl of diluted semen was pipetted into a pre-warmed 20 µm-depth counting chamber and sperm motility was assessed within 20 sec.

For each sample multiple microscope fields were analyzed. Some parameters were measured directly on the digital images (velocity parameters and movements of the head) whilst others were calculated from the measurements, *e.g*. the straightness of movement and the percentage of motile or progressively motile cells. The cell track was reconstructed on sequential digital images by the accompanying software.

The measured parameters used were: curvilinear velocity (VCL, µm/s), average path velocity (VAP, µm/s), straight line velocity (VSL, µm/s), amplitude of lateral head displacement (ALH, µm), linearity (LIN, %), and straightness (STR).

### Fluorescent multiple staining (FMS)

Sperm plasma membrane integrity was assessed using FMS.

Fluorochromes used in this study were:

- Propidium Iodide (PI) a red-fluorescent nuclear and chromosome counterstain. Since **propidium iodide** does not penetrate live cells, it is commonly used to identify dead cells in a population. To make a stock solution the solid form was dissolved in deionized water to a concentration of 1 mg/mL (1.5 m*M*).
- Fluorescein isothiocyanate-conjugated peanut agglutin (FITC-PNA) was used to determine the acrosome status of viable spermatozoa. FITC-PNA intensely labels the acrosome region of acrosome-reacted spermatozoa. A stock solution was prepared by dissolving 2 mg of FITC-PNA in 1 mL PBS pH 7.4.
- 5,5’,6,6’-tetrachloro-1,1’,3,3’ tetraethylbenzimidazolyl-carbocyanine iodide (JC-1) is a selective mitochondrial stain. Cells with a high membrane potential form J-aggregates, thus showing high red fluorescence with JC-1. In cells with a low membrane potential JC-1 maintains (or re-acquires) its monomeric form, thus showing only green fluorescence. A stock solution of 0.153 m*M* in dimethylsulfoxide (DMSO) was prepared.

The protocol of multiple fluorescent staining was as follow: samples of semen were diluted to a concentration 15 × 10^6^ spermatozoa/mL with HEPES/BSA solution (130 m*M* NaCl, 4 m*M* potassium chloride, 14m*M* fructose, 10m*M* HEPES, 1m*M* calcium chloride, 0.5 m*M* magnesium chloride, 0.1% BSA), then 500 µL of diluted semen was incubated with 2 µL JC-1 at 37°C for 30 min under light-proof conditions.

At the end of the first incubation 2.5 µL PI and 2.5 µL FITC-PNA were added and the second incubation was performed at 37°C for 5 min under light-proof conditions. Then, 30 µL of 10% formalin solution was added to fix cells. Three hundred spermatozoa per slide were examined immediately by microscope with Olympus BX 51 and 100x objective using a simultaneous combination of excitation and emission filters at 488/650 nm.

**Fig. 1:**
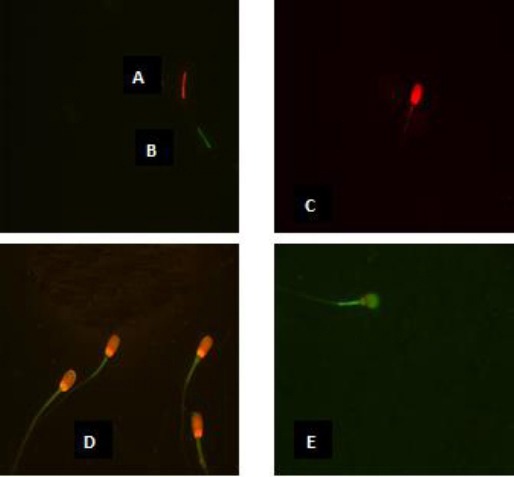
Spermatozoa viewed under phase contrast microscopy: spermatozoa alive and intact (colorless head) with high mitochondrial membrane potential (A); spermatozoa alive and intact (colorless head) with low mitochondrial membrane potential (B); dead spermatozoa (red head) with high mitochondrial membrane potential (C); dead spermatozoa (red head) with low mitochondrial membrane potential (D); reacted dead spermatozoa (red and green head) with low mitochondrial membrane potential (E).

By combining the individual stains of each of the fluorochromes, at the end of FMS different categories of spermatozoa were obtained as listed in Table 2 and shown in Fig. 1.

**Table 2:** Spermatozoa categories after fluorescent multiple staining.

Category	Description
Colourless head/red mitochondria	Living and undamaged spermatozoa with high membrane potential.
Colourless-green head/red mitochondria	Living spermatozoa with high membrane potential but acrosome reaction.
Colourless head/green mitochondria	Living and undamaged spermatozoa with low membrane potential.
Colourless-green head/green mitochondria	Living spermatozoa with low membrane potential but acrosome reaction (hypothetical combination).
Red head/red mitochondria	Destabilized spermatozoa with high membrane potential.
Red-green head/red mitochondria	Destabilized spermatozoa with high membrane potential and acrosome reaction.
Red head/green mitochondria	Dead spermatozoa with low membrane potential.
Red-green head/green mitochondria	Dead spermatozoa with low membrane potential and acrosome reaction.

### Thermoresistance test

Samples of fresh semen diluted in each extender were incubated in a water bath at 37°C and, for each extender, an aliquot was taken every 2 hours for assessment of the parameters of motility of the spermatozoa.

### Microbiological test

Bacteriological status of both fresh and diluted samples was evaluated by CFU. One aliquot from each these samples was analyzed immediately and the other after 6 days of storage at 19°C. In order to identify CFU, the samples were seeded by means of a spatulate technique in a TSA (Tryptone Soy Agar) plate and incubated for 18-24 hours at 37°C. The identification of isolates was then performed using API-system (Poli *et al.*, 2005).

### Statistical analysis

All the experiments were repeated three times with pooled ejaculates from the three boars. The data reported represent the mean values. Data were first tested using a Kolgomorov–Smirnof test to determine the normality of their distribution. In view of the Gaussian distribution of the data, a system of linear model of ANOVA (Software Stat Tools by Palisade EMEA, West Drayton Middlesex, UK) was used to compare the differences of motility parameters and patterns of multiple fluorescent staining within each extender. Pair-wise comparisons between the groups were made based on Tukey’s *post hoc* test. Means were considered significantly different with a p-value <0.05. Moreover, a study on the correlation (Pearson) between the total percentage of motile spermatozoa and motility parameters and between percentage of motile spermatozoa and result of fluorescent multiple staining was performed. The test conducted was a simple linear regression with the software Minitab 1.4 (Minitab Inc., State College, PA, USA). Regressions were considered highly significant with coefficient of determination (r^2^) greater than 0.9.

## Results

### Extender analysis

The results of pH and osmolality analysis of the extenders are shown in Table 3. The pH ranged from 6.70 to 7.39 and osmolality ranged from 241 to 331 Osm/Kg.

**Table 3:** pH and osmolality of extenders used in experiment

Extender	pH (at 37°or 20°C)	Osm/Kg (at 37°/20°C)
MR-A 3	6.91 / 6.96	241 / 245
Androstar - Plus	6.93 / 6.91	279 / 269
BTS	6.97 / 6.96	325 / 327
Modena 1	6.98 / 7.00	286 / 305
ZM	7.09 / 7.08	330 / 335
Mr Pig Sire	7.39 / 7.44	331 / 325
Sus	6.43 / 6.44	263 / 277
Tri-X Cell	6.70 / 6.75	291 / 296
HP2000	6.96 / 6.93	284 / 283

### Assessment of fresh boar sperm

The boars used in this study were high quality breeding animals as demonstrated by their average return rate that was 9.5% which was not significantly different from the mean value of Provincial Breeders Association (10.84%).

The quality-quantitative analysis of spermatozoa was carried out by classical methods and the sperm samples analyzed met the minimum criteria (volume of spermatozoa-rich fraction > 100 mL, sperm concentration in ejaculate > 200 × 10^6^ cells/mL, motility >70%, normal sperm morphology >80%) to be considered of good quality for AI.

### Motility assessment using CASA

The percentage of motile spermatozoa decreased with storage time (statistical significance *P*<0.05), (Tables 4a and 4b). The percentage of motile cells remained higher in extenders BTS and TRI-X-Cell (short and long-term respectively) with values 55.95 ± 1.89% and 36.56 ± 3.46%.

**Table 4a:** Evaluation of sperm motility in different short-term extenders with CASA system

Short term extender	Days	Concentration (x 10^6^/mL)	Motile spermatozoa (%)	Progressive spermatozoa (%)	VCL (µm/s)^1^	VSL (µm/s)^1^	VAP (µm/s)^1^	LIN (%)^1^	STR^1^	AHL (µm)^1^
MR-A 3	0	50.39±1.19^a^	92.10±2.49^a^	1639±1.36^a^	78.41±4.25^a^	38.16±4.06^a^	49.59±2.52^a^	48.66±1.73^a^	0.61±0.01^a^	6.13±0.98^a^
	2	53.5±2.35^a^	64.82±3.84^b^	5.18±1.36^b^	65.51±4.05^b^	24.84±2.53^b^	43.27±4.02^b^	37.91±0.95^b^	0.62±0.01^b^	6.77±0.59^a^
	4	51.75±2.00^a^	42.39±2.74^c^	4.45±0.52^c^	64.40±3.81^c^	24.19±1.19^b^	45.67±2.20^b^	37.56±1.72^c^	0.57±0.04^c^	7.12±0.13^b^
	6	49.85±1.97^a^	23.21±1.52^d^	0.64±0.31^d^	45.46±3.89^d^	10.78±2.15^c^	31.32±3.01^c^	23.71±2.19^d^	0.44±0.03^d^	7.18±0.68^b^
Androstar	0	49.30±0.04^a^	90.58±3.21^a^	15.89±1.04^a^	77.63±0.66^a^	34.88±1.18^a^	45.03±3.89^a^	44.93±2.17^a^	0.62±0.01^a^	5.50±0.37^a^
	2	49.71±2.25^a^	68.37±3.07^b^	5.30±1.90^b^	69.21±2.07^b^	24.66±3.25^b^	32.41±1.05^b^	35.63±2.52^b^	0.59±0.01^b^	6.34±0.15^b^
	4	51.59±1.09^a^	35.35±2.25^c^	4.51±1.05^c^	61.12±2.68^c^	19.44±2.36^c^	40.17±1.67^c^	31.80±2.36^b^	0.53±0.02^c^	6.50±0.40^bc^
	6	50.27±0.68^a^	21.96±2.08^d^	0.55±0.35^d^	48.19±2.11^d^	10.01±2.41^d^	23.14±2.57^d^	20.77±2.34^b^	0.52±0.02^d^	6.86±0.36^bc^
Mr Pig Sire	0	50.85±0.68^a^	90.45±1.70^a^	14.95±0.89^a^	82.28±4.51^a^	36.06±0.93^a^	46.75±2.80^a^	43.82±1.12^a^	0.62±0.02^a^	6.39±0.30^a^
	2	51.01±1.44^a^	66.17±4.51^b^	4.49±0.45^b^	68.34±2.60^b^	17.22±0.70^b^	27.78±2.13^b^	25.19±1.67^b^	0.64±0.01^b^	6.41±0.14^a^
	4	51.05±0.77^a^	43.36±2.34^c^	0.48±0.37^c^	48.50±5.63^c^	14.16±2.16^c^	26.94±2.23^b^	29.19±1.74^c^	0.65±0.00^c^	7.62±0.32^b^
	6	50.97±2.53^a^	20.17±2.14^d^	0.00±0.00^d^	22.78±4.45^d^	4.12±0.81^d^	14.19±1.73^c^	18.08±1.82^d^	0.65±0.01^c^	7.76±0.48^b^
ZM	0	50.26±0.75^a^	91.97±5.13^a^	16.25±0.59^a^	74.96±3.46^a^	37.89±1.92^a^	44.03±2.52^a^	50.54±2.18^a^	0.62±0.01^a^	5.50±0.43^a^
	2	49.18±2.52^a^	38.14±3.22^b^	4.16±0.81^b^	26.69±3.46^b^	26.42±5.43^b^	36.01±3.35^b^	98.98±0.65^b^	0.61±0.01^b^	5.72±0.34^a^
	4	50.95±0.98^a^	13.33±1.56^c^	2.26±0.53^c^	22.79±3.37^c^	4.16±0.35^c^	6.21±0.87^c^	18.25±2.30^c^	0.61±0.01^b^	5.64±0.80^ab^
	6	49.92±2.32a	3.83±0.84^d^	0.00±0.00^d^	19.21±3.19^d^	3.40±0.63^c^	5.88±0.56^c^	17.69±0.40^c^	0.62±0.01^a^	6.50±0.44^b^
1Modena	0	52.30±0.43^a^	92.04±0.80^a^	15.64±0.42^a^	79.57±3.50^a^	36.76±2.36^a^	46.57±4.91^a^	46.19±0.82^a^	0.63±0.01^a^	6.61±0.54^a^
	2	51.25±2.07^a^	54.29±3.52^b^	5.43±0.68^b^	55.15±4.97^b^	20.06±1.05^b^	31.69±2.04^b^	36.37±2.80^b^	0.62±0.01^b^	6.88±0.59^a^
	4	50.93±0.21^a^	21.33±3.10^c^	3.97±0.18^c^	41.08±2.18c	19.26±1.17^c^	30.07±3.74^b^	46.88±2.04^c^	0.62±0.01^b^	7.22±0.21^b^
	6	50.33±1.00^a^	16.62±1.79^d^	0.00±0.00^d^	26.07±3.37^d^	5.59±0.60^d^	7.89±1.38^c^	21.44±2.19^c^	0.62±0.01^b^	7.43±0.45^b^
BTS	0	51.36±2.31^a^	91.29±1.43^a^	15.69±1.23^a^	88.44±2.66^a^	39.17±6.74^a^	46.28±4.19^a^	44.28±1.54^a^	0.71±0.01^a^	4.26±0.37^a^
	2	50.42±1.74^a^	68.13±4.21^b^	6.11±0.78^b^	77.66±6.56^b^	26.14±2.01^b^	41.66±2.24^b^	33.65±2.39a^b^	0.71±0.01^a^	5.75±0.37^b^
	4	50.98±0.76^a^	61.78±2.60^c^	3.92±0.29^c^	68.50±3.45^c^	20.80±4.75^c^	42.71±5.41^b^	30.36±2.35^abc^	0.71±0.01^a^	6.46±0.49^c^
	6	50.69±1.18^a^	55.95±1.89^d^	2.57±0.33^c^	54.12±3.42^d^	14.13±1.39^d^	27.58±1.32^c^	26.10±1.04^bc^	0.70±0.01^b^	6.85±0.36^c^

^a,d^ Means (± SE) within a column not sharing a common superscript differ (*P* < 0.05).

1 VCL = curvilinear velocity (µm/s); VSL = straight line velocity (µm/s); VAP = average path velocity (µm/s). LIN = linearity, ratio in percentage of VSL/VCL (%); STR = straightness, VSL/VAP; ALH = amplitude of lateral head displacement (µ).

**Table 4b:** Evaluation of sperm motility in different long-term extenders with CASA system

Long-term extender	Days	Concentration (x 10^6^/mL)	Motile spermatozoa (%)	Progressive spermatozoa (%)	VCL (µm/s)^1^	VSL (µm/s)^1^	VAP (µm/s)^1^	LIN (%)^1^	STR^1^	AHL (µm)^1^
Tri-X-Cell	0	50.59±0.73^a^	91.11±1.63^a^	16.66±1.38^a^	102.95±2.46^a^	47.23±3.02^a^	65.14±5.54^a^	45.87±1.24^a^	0.67±0.02^a^	5.96±0.47^a^
	2	51.84±1.81^a^	69.46±5.96^b^	8.66±0.51^b^	98.51±4.79^b^	34.84±1.19^b^	60.73±2.35^b^	35.36±1.54^b^	0.67±0.01^a^	6.23±0.12^b^
	4	50.52±1.24^a^	62.34±4.53^c^	5.46±0.39^c^	84.94±4.66^c^	25.04±0.95^c^	50.55±4.01^c^	29.48±2.38^c^	0.66±0.01^b^	6.33±0.17^b^
	6	50.16±1.77^a^	54.67±3.59^d^	3.93±0.37^d^	48.82±0.95^d^	14.35±1.73^d^	25.62±3.80^d^	29.39±2.56^bc^	0.66±0.02^b^	6.41±0.41^b^
	8	50.55±0.76^a^	52.04±3.31^d^	1.29±0.46^e^	46.28±3.45^d^	15.90±2.03^d^	22.72±2.79^d^	34.35±1.24^b^	0.66±0.01^b^	6.47±0.35^b^
	10	49.86±0.54^a^	48.39±3.24^d^	1.61±0.19^e^	43.72±2.33^d^	10.59±2.03^e^	16.91±2.20^e^	24.22±3.07^b^	0.64±0.01^c^	6.57±0.35^b^
	12	50.76±1.22^a^	36.56±3.46^e^	0.53±0.31^f^	32.70±1.28^e^	6.58±1.22^f^	16.87±2.62^e^	20.12±0.63^c^	0.64±0.01^c^	6.82±0.38^b^
HP2000	0	49.77±1.84^a^	91.04±1.20^a^	16.50±1.12^a^	84.27±5.22^a^	35.62±3.33^a^	47.55±2.13^a^	42.27±3.11^a^	0.62±0.01^a^	5.56±0.23^a^
	2	51.31±0.79^a^	74.67±2.47^b^	5.54±0.51^b^	69.45±1.64^b^	21.90±1.16^b^	38.33±1.09^b^	31.53±0.82^b^	0.61±0.01^b^	5.59±0.39^a^
	4	50.71±1.64^a^	55.88±3.54^c^	2.57±0.15^c^	57.20±2.08^c^	15.02±0.80^c^	27.21±1.64^c^	26.26±2.27^b^	0.60±0.02^c^	5.67±0.31^a^
	6	50.72±2.70^a^	45.93±3.32^d^	1.67±0.15^c^	48.14±1.71^d^	12.70±0.97^d^	25.90±1.33^c^	26.38±2.30^b^	0.61±0.01^b^	7.42±0.18^b^
	8	50.99±2.24^a^	44.73±4.40^d^	1.57±0.20^c^	35.96±4.42^e^	11.21±1.63^d^	20.58±1.01^d^	31.17±2.43^b^	0.62±0.01^a^	7.45±0.38^b^
	10	49.98±1.25^a^	32.39±3.19^e^	0.53±0.11^d^	30.74±2.78^f^	7.61±1.20^e^	15.94±1.04^e^	24.75±3.02^c^	0.62±0.01^a^	7.51±0.17^b^
	12	51.19±0.31^a^	25.63±2.49^f^	0.00±0.00^e^	31.94±2.98^f^	6.32±0.46^e^	15.82±2.08^e^	19.78±2.15^c^	0.60±0.01^c^	7.59±0.30^b^
Sus	0	51.67±0.62^a^	88.43±5.32^a^	16.37±0.49^a^	71.91±2.19^a^	34.94±3.14^a^	45.64±1.30^a^	48.58±2.59^a^	0.61±0.02^a^	5.20±0.59^a^
	2	50.45±1.37^a^	65.76±3.29^b^	5.46±0.37^b^	65.08±4.16^b^	20.27±1.02^b^	34.83±2.54^b^	31.14±1.97^b^	0.62±0.01^b^	5.42±0.45^b^
	4	50.73±1.11^a^	44.79±3.88^c^	0.50±0.10^c^	57.51±1.58^c^	12.63±1.21^c^	26.67±2.89^c^	21.96±1.49^c^	0.58±0.01^c^	5.61±0.42^b^
	6	50.90±0.85^a^	35.74±4.09^d^	0.00±0.00^d^	57.08±5.23^c^	11.43±1.48^c^	25.95±2.00^c^	20.02±1.84^d^	0.57±0.02^d^	5.85±0.09^c^
	8	49.54±1.18^a^	32.62±1.21^d^	0.00±0.00^d^	42.72±2.19^d^	9.00±0.36^d^	16.92±1.08^d^	21.07±0.49^d^	0.57±0.01^d^	5.91±0.10^c^
	10	48.97±1.50^a^	18.64±3.65^e^	0.00±0.00^d^	44.17±2.78^d^	9.55±1.58^d^	16.62±2.06^d^	21.62±2.07^e^	0.57±0.02^d^	7.64±0.37^d^
	12	50.78±0.56^a^	14.50±2.74^e^	0.00±0.00^d^	23.63±4.07^e^	5.13±0.21^e^	9.19±1.28^e^	21.71±2.94^e^	0.56±0.02^e^	7.64±0.36^d^

^a,f^ Means (± SE) within a column not sharing a common superscript differ (*P* < 0.05).

1 VCL = curvilinear velocity (µm/s); VSL = straight line velocity (µm/s); VAP = average path velocity (µm/s), LIN = linearity, ratio in percentage of VSL/VCL (%); STR = straightness, VSL/VAP; ALH = amplitude of lateral head displacement (µ).

The minimum value of 60% of motility (Britt *et al.*, 1999) in short and long-term extenders are guaranteed until to fourth day in both extenders. From this analysis there appeared to be a low rate of progressive spermatozoa after day 0 of storage in all extenders tested (ranging from 14.95 ± 0.89% in MR Pig Sire to 16.66 ± 1.38% in Tri-X-Cell). This may be the result of dilution shock which is known to decrease progressive motility.

**Fig. 2:**
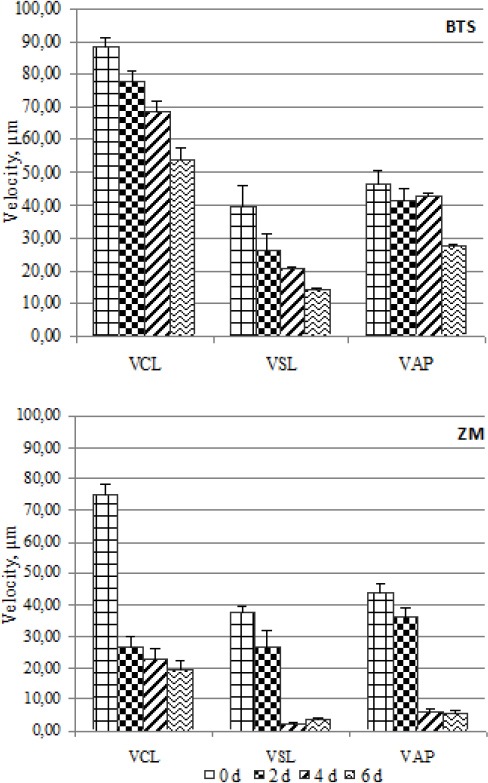
Comparison between best (BTS) and worst (ZM) short-term extender. Data are represented as mean±SEM

**Fig. 3:**
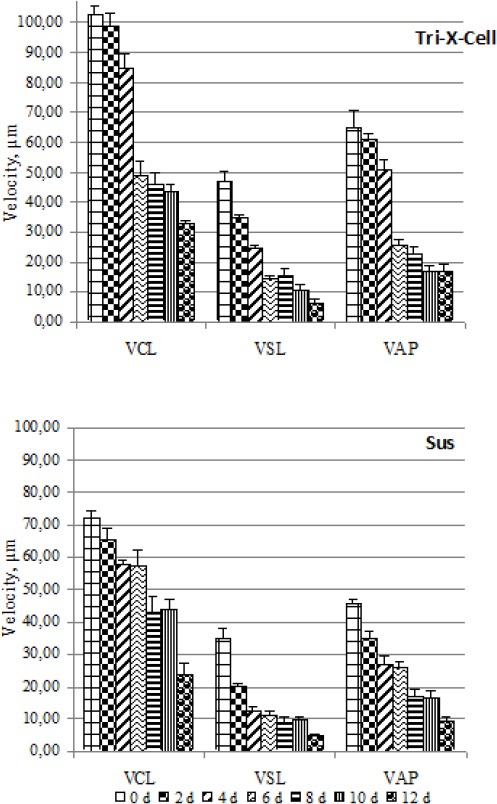
Comparison between best (Tri-X-Cell) and worst (Sus) long-term extender. Data are represented as mean±SEM

**Fig. 4:**
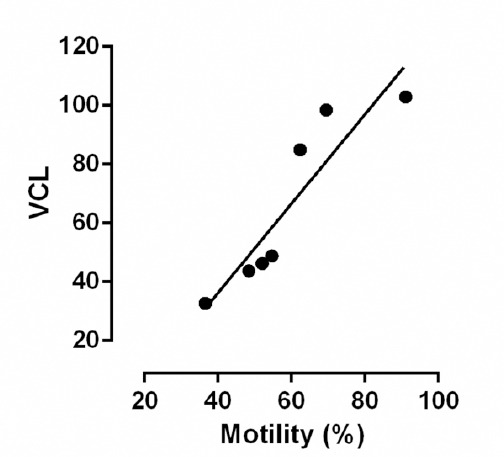
Correlation between rate of motility and VCL in Tri-X-Cell.

At the end of storage the values of velocity (VCL, VAP and VSL), the percentage of LIN and the value of STR were significantly higher (*P* <0.05) in extenders BTS (short-term) and Tri-X-Cell (long-term). Motility values of spermatozoa in BTS were compared with those of ZM, which was considered to be the worst short-term extender, and the motility values of extender Tri-X-Cell were compared with those of extender Sus, considered to be the worst long-term extender. These comparisons are presented in Figs. 2 and 3. Data from the analysis of the correlation between the percentage of motile spermatozoa and the parameters of motility detected by the CASA system showed that the VCL values had a high correlation (r^2^ > 0.9) with the percentage of motility in almost all extenders, as showed in the Figure 4 for Tri-X-Cell extender, whilst the VSL generally showed a low correlation (r^2^ ≤ 0.8).

### Fluorescent multiple staining (FITC-PNA/PI/JC-1)

All cells with colourless heads were classified as viable spermatozoa regardless of the potential of mitochondrial membrane. No living spermatozoa were identified with a low membrane potential and acrosome reaction, so this category was considered a hypothetical combination.

As shown in Tables 5a and 5b, initial sperm viability was approximately 90% for all extenders. Of the short-term extenders, BTS provided the best preservation of living spermatozoa (52.67 ± 3.51%), after 6 days of storage, and deviated significantly (*P* <0.05) from the other extenders where the percentage of living spermatozoa ranged between 1.67% and 20.67%. Extender Tri-X-Cell performed best amongst the long-term extenders with 35.67 ± 1.15% viable cells after 12 days of storage at 19°C compared to the other two extenders where the viable cells ranged between 14.50% and 25.63%.

**Table 5a:** Percentage of spermatozoa belong to different categories highlighted by triple staining technique.

	Short-term extender						
Category	Days	MR-A 3	Androstar	Mr Pig Sire	ZM	Modena 1	BTS
Living and undamaged spermatozoa with high membrane potential	0	89.00± 5.57^a^	88.00±8.19^a^	88.67±2.52^a^	88.00±2.00^a^	88.33±2.52^a^	89.67±4.04^a^
	2	63.67± 1.53^a^	67.67±3.51^a^	65.00±3.61^a^	39.67±6.03^b^	50.33±1.53^c^	67.33±4.93^c^
	4	43.67± 1.53^a^	34.00±1.00^b^	40.33±3.21^c^	15.33±2.52^d^	20.67±3.79^e^	58.33±3.06^f^
	6	20.33± 4.04^a^	20.67±1.53^a^	19.67±3.79^a^	1.67±0.58^b^	15.33±2.31^c^	52.67±3.51^d^
Living spermatozoa with high membrane potential but acrosome reaction	0	1.00±0.00^a^	0.33±0.58^a^	0.00±0.00^b^	1.00±0.00^a^	0.67±0.58^c^	1.33±0.58^d^
	2	0.33±0.58^a^	0.33±0.58^a^	0.00±0.00^b^	0.33±0.58^a^	0.00±0.00^b^	0.00±0.00^b^
	4	1.00±1.00^a^	0.33±0.58^b^	0.00±0.00^c^	0.00±0.00^c^	0.00±0.00^c^	1.00±1.00^a^
	6	0.67±0.58^a^	0.33±0.58^b^	0.00±0.00^c^	0.33±0.58^a^	0.00±0.00^c^	0.33±0.58^a^
Living and undamaged spermatozoa with low membrane potential	0	0.00±0.00^a^	0.00±0.00^a^	0.00±0.00^a^	0.00±0.00^a^	0.00±0.00^a^	0.00±0.00^a^
	2	0.00±0.00^a^	0.00±0.00^a^	0.67±0.58^b^	0.67±0.58^b^	1.00±1.00^b^	0.00±0.00^a^
	4	3.00±2.65^a^	0.00±0.00^b^	3.33±2.08^a^	1.33±0.58^c^	1.33±1.15^c^	1.67±0.58^c^
	6	2.67±1.15^a^	0.00±0.00^b^	1.33±0.58^c^	1.00±1.00^c^	1.67±0.58^c^	1.00±1.00^c^
Destabilized spermatozoa with high membrane potential	0	5.33±1.53^ac^	6.33±2.52^a^	6.67±2.08^a^	1.33±1.53^b^	4.33±1.53^ac^	3.33±1.53^c^
	2	15.33±1.53^a^	14.33±0.58^a^	7.67±3.21^b^	16.67±2.08^a^	9.00±2.65^b^	9.33±3.51^b^
	4	14.33±2.08^a^	13.33±1.15^ab^	11.00±2.65^b^	14.67±2.31^a^	16.33±1.53^a^	8.00±3.00^c^
	6	15.67±1.53^a^	14.00±3.00^a^	17.00±1.73^a^	14.00±2.65^a^	16.33±1.53^a^	9.00±4.00^b^
Destabilized spermatozoa with high membrane potential and acrosome reaction	0	1.33±0.58^a^	2.33±1.15^b^	2.00±1.00^b^	4.33±2.08^c^	2.33±1.53^b^	1.67±0.58^ab^
	2	7.00±2.00^a^	8.67±1.15^a^	6.67±0.58^a^	13.00±1.73^b^	4.33±2.52^c^	4.67±2.08^c^
	4	11.33±2.08^a^	10.67±1.53^a^	10.00±3.46^a^	14.33±2.52^b^	7.67±0.58^c^	7.00±2.00^c^
	6	14.00±2.65^a^	5.33±1.53^b^	11.33±1.53^c^	9.00±2.65^c^	8.00±2.00^d^	4.00±1.73^b^

^a,f^ Means (± SE) within a line not sharing a common superscript differ (*P* < 0.05).

**Table 5b:** Percentage of spermatozoa belong to different categories highlighted by triple staining technique.

	Long-term extender				
Category		Days	Tri-X-Cell	HP2000	Sus
Living and undamaged spermatozoa with high membrane potential		0	87.33±4.93^a^	86.00±2.00^a^	83.33±6.51^a^
		2	67.00±3.00^a^	72.33±6.66^b^	64.00±3.00^a^
		4	58.33±4.04^af^	54.67±3.21^a^	45.33±4.04^b^
		6	52.67±4.04^a^	45.00±4.00^b^	33.67±3.51^c^
		8	50.33±2.52^a^	42.33±2.08^b^	32.67±1.53^c^
		10	46.00±3.00^a^	30.00±2.65^b^	18.33±4.51^c^
		12	35.67±1.15^a^	22.67±2.08^b^	15.33±1.53^c^
Living spermatozoa with high membrane potential but acrosome reaction		0	0.33±0.58^a^	0.33±0.58^a^	0.00±0.00^b^
		2	0.00±0.00^a^	0.00±0.00^a^	0.33±0.58^b^
		4	0.67±0.58^a^	0.00±0.00^b^	0.00±0.00^b^
		6	0.00±0.00^a^	0.33±0.58^b^	0.00±0.00^a^
		8	0.00±0.00^a^	0.00±0.00^a^	0.00±0.00^a^
		10	0.00±0.00^a^	0.00±0.00^a^	0.00±0.00^a^
		12	0.00±0.00^a^	0.00±0.00^a^	0.00±0.00^a^
Living and undamaged spermatozoa with low membrane potential		0	0.00±0.00^a^	0.00±0.00^a^	0.00±0.00^a^
		2	1.33±1.15^a^	0.33±0.58^b^	0.00±0.00c
		4	0.00±0.00^a^	1.33±1.15^b^	0.00±0.00^a^
		6	0.67±0.58^a^	0.67±0.58^a^	0.00±0.00^b^
		8	0.00±0.00^a^	0.00±0.00^a^	0.00±0.00^a^
		10	0.00±0.00^a^	0.33±0.58^b^	0.00±0.00^a^
		12	0.00±0.00^a^	0.33±0.58^b^	0.00±0.00^a^
Destabilized spermatozoa with high membrane potential and acrosome reaction		0	8.67±1.15^a^	8.00±3.00^a^	8.33±2.08^a^
		2	16.67±4.04^a^	13.33±1.53^a^	14.33±1.53^a^
		4	15.33±2.08^a^	13.00±1.73^a^	12.33±1.53^a^
		6	15.00±1.73^a^	13.67±2.52^a^	12.33±1.53^a^
		8	14.00±3.00^a^	14.33±1.15^a^	10.33±1.53^b^
		10	8.67±1.15^a^	15.33±2.52^b^	6.67±0.58^c^
		12	5.67±1.15^a^	9.67±3.21^b^	5.00±1.73^a^
Destabilized spermatozoa with high membrane potential and acrosome reaction		0	1.00±1.00^a^	1.33±0.58^a^	2.00±1.73^a^
		2	4.67±0.58^a^	4.33±0.58^a^	5.00±2.65^a^
		4	6.00±2.65^a^	7.67±1.15^a^	8.33±1.15^a^
		6	8.00±2.65^a^	7.67±3.5^a^	7.33±1.15^a^
		8	9.00±1.00^a^	9.67±2.31^a^	7.67±0.58^b^
		10	11.00±2.65^a^	10.00±1.73^a^	5.67±1.15^b^
		12	4.67±1.53^a^	5.33±1.53^a^	5.67±1.15^a^
Dead spermatozoa with low membrane potential		0	2.33±0.58^a^	3.67±1.53a^b^	4.33±0.58a^b^
		2	7.33±1.53^a^	8.67±3.06a^b^	10.33±2.89a^b^
		4	13.00±3.61^a^	13.67±2.52^a^	21.33±1.53^b^
		6	16.33±1.53^a^	19.67±1.15^b^	30.67±3.21^c^
		8	18.00±2.00^a^	20.00±3.61^a^	26.33±5.57^b^
		10	23.00±2.00^a^	26.00±2.00^b^	41.33±3.06^c^
		12	30.33±1.53^a^	36.00±3.00^b^	42.67±3.21^c^
Dead spermatozoa with low membrane potential and acrosome reaction		0	0.67±0.58^a^	1.33±0.58^b^	2.33±0.58^c^
		2	2.33±0.58^a^	3.33±1.53^a^	6.67±1.15^b^
		4	6.67±1.15^a^	10.33±2.89^b^	13.33±2.08^b^
		6	7.67±2.52^a^	13.67±1.15^b^	16.00±2.65^b^
		8	9.00±1.73^a^	14.67±3.06^b^	23.00±2.00^c^
		10	11.33±3.06^a^	18.67±3.21^b^	28.00±2.65^c^
		12	24.67±5.13^a^	26.67±2.08^b^	32.00±4.00^c^

^a,c^ Means (± SE) within a line not sharing a common superscript differ (*P* < 0.05).

**Fig. 5:**
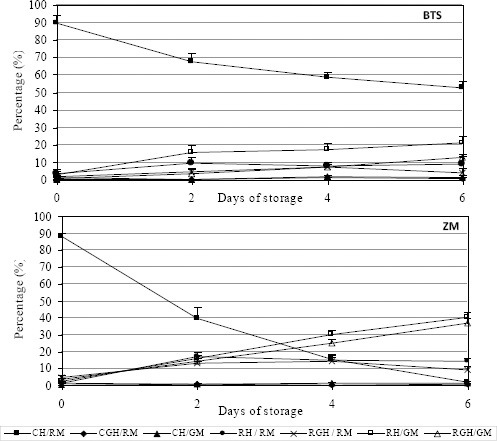
Comparison between best (BTS) and worst (ZM) short-term extender. Data are represented as mean±SEM.

**Fig. 6:**
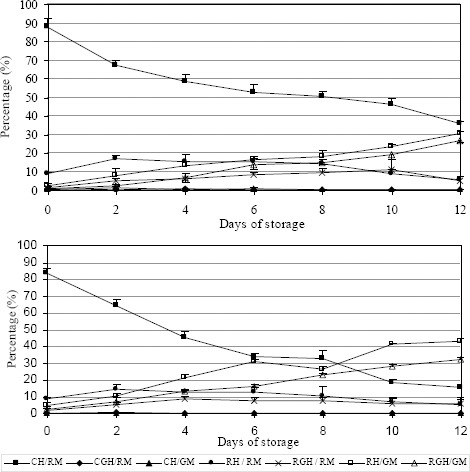
Comparison between best (Tri-X-Cell) and worst (Sus) long-term extender. Data are represented as mean±SEM.

In the best and worst short-term (BTS and ZM) and long-term (Tri-X-Cell and Sus) extenders both the percentage of viable spermatozoa, and the numbers of nonviable spermatozoa with low membrane potential and/or acrosome reaction (red head/green mitochondria and red-green head/green mitochondria) decrease physiologically and significantly (*P* <0.05) with duration of storage (Figs. 5 and 6). There were few viable spermatozoa with a spontaneous acrosomal reaction in all tested extenders and at different days of storage. Typically more than 99% of viable spermatozoa had intact acrosomes.

The number of undamaged spermatozoa, recorded with FMS, has been positively correlated with the total percentage of motile spermatozoa obtained using CASA and the correlation was high in all analyzed extenders (r^2^ was greater than 0.9).

There were two very interesting categories of spermatozoa that stained positively with propidium iodide and had a high mitochondrial membrane potential and/or were positive with FITC-PNA (red head/red mitochondria, red-green head/red mitochondria): these spermatozoa were termed “destabilized”.

As storage time increased the percentage of live spermatozoa decreased and the percentage of destabilized cells (with red head/red mitochondria and red-green head/red mitochondria) increased. These cells were not considered ‘dying’ because they had mitochondria with a high membrane potential. With respect to destabilized cells with intact acrosomes, extenders BTS and Tri-X-Cell were again the best performing short and long-term extenders respectively because they had the lowest numbers of destabilized cells.

**Fig. 7:**
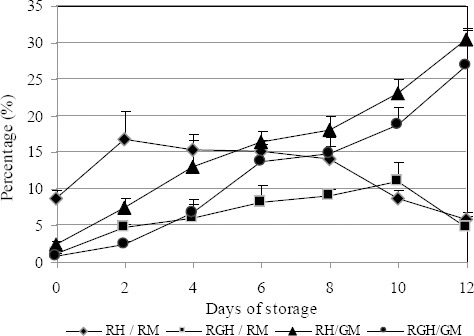
Development of destabilized and dead cells with continuing preservation in extender Tri-X-Cell. Data are represented as mean±SEM. Legend: RH/RM = Red head / Red mitochondria; RGH/RM = Red green head / Red mitochondria; RH/GM = Red head / green mitochondria; RGH/GM = Red green head / green mitochondria.

The number of destabilized acrosome reacted cells was lowest in BTS but there was no significant differences between the long-term extenders at the end of storage. Statistical analysis showed that, in almost all studied extenders, the percentage of destabilized cells decreased as numbers of dead cells increased in the last days of storage. This is clearly shown for Tri-X-Cell in Fig. 7.

The percentage of dying spermatozoa increased during storage in all extenders with a sharp increase occurring between the fourth and sixth day of storage for short-term extenders and between the tenth and twelfth day of storage for long-term extenders. Once again extenders BTS and Tri-X-Cell provided better preservation of the viability of spermatozoa.

### Thermoresistance test

**Fig. 8:**
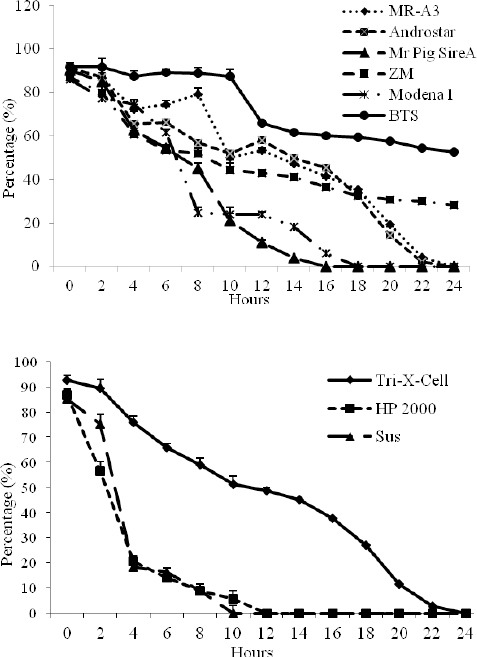
Percentage of motility during thermoresistance test in short and long-term extenders. Data are represented as mean±SEM

In all samples, motility reduction was significant (*P*< 0.05) and gradual over time, although more continuous in extender Tri-X-Cell. In extender BTS, after 24 hours of incubation at 37°C, the percentage of motile cells was 52.56 ± 2.04%. In this test, extenders BTS and Tri-X-Cell appeared superior in preservation of survival of spermatozoa at the physiological temperature characteristic of female genitals. The results of the thermoresistance test are presented in Fig. 8.

### Microbiological test

CFU numbers remained stable during the study period and values were estimated to be around 10^5^-10^6^ CFU/mL in all extenders. Bacterial contamination was characterized by environmental contaminants, in particular Gram-negative and oxidase positive bacteria: *Pseudomonas fluorescens, Aeromonas sobria, Ochrobactrum anthropi*.

There was no significant correlation between CFU, motility and numbers of dead spermatozoa.

## Discussion

There has been an exponential increase in the use of AI in the swine industry worldwide. Most boar semen is diluted and stored at temperatures slightly below room temperature (17-20°C), although a certain percentage is also deep-frozen for gene-banking purposes or for gene export. Consequently, preservation of the fertilizing capacity of boar semen for several days remains an important target for the industry. The commercial extenders are specific for short or long-term preservation but during storage spermatozoa may undergo changes that involve all their structures. Motility is considered an important parameter in assessment of the quality of boar spermatozoa (Britt *et al.*, 1999; Johnson *et al.*, 2000). In this study sperm motility was affected by duration of storage and the extender used.

The percentage of motile spermatozoa decreased in all extenders as storage time increased and this could be correlated with variation in oxygen uptake and metabolic activity depending on the extender used. The percentage of motile cells was higher in extenders BTS and Tri-X-Cell (short- and long-term respectively) when compared with the other extenders, but the minimum value of 60% motility (Britt *et al.*, 1999) is only guaranteed until the fourth and sixth days in short and long-term extenders respectively.

This study reconfirms that short-term extenders assure at least 3 days of preservation but the long-term extenders used in this study are not suitable for a preservation period of more than 5-6 days. These data agree with De Ambrogi *et al*. (2006) that reported a significant decrease in motility from 72 hours for MR-A and BTS extended semen and at 96 hours for X-Cell-extended semen. Also Vyt *et al*. (2004) observed a significant decrease in motility in boar semen extended with five different extenders, but in their trial there was a higher motility in semen extended with BTS than with a long-term extender (Androhep). This is in contrast with other studies where semen extended in BTS showed lower motility in comparison with spermatozoa suspended in a long-term extender (Huo *et al.*, 2002; Kommisrud *et al.*, 2002; Dubé *et al.*, 2004).

We didn’t compare short vs long-term extender because we studied different time of preservation: until day 6 for short and until day 12 for long extenders, but if the rate of motility at the day six, for both kinds of extenders, is compared it is possible to observe no difference in the decrease of motility between BTS and Tri-X-cell.

This result obviously underline that a long extender preserves spermatozoa for short time in the same way of short extender.

From our results it appears that there was an insufficient percentage of progressive spermatozoa from day 0 of storage in all the extenders tested. This may be due to dilution shock which decreases progressive motility and increases circular motility. The sperm motility in boars is very dependent on the environment, and factors like temperature and the extender used, or even the type and size of slides and cover slips used, can influence it greatly (Quintero-Moreno *et al.*, 2004; Vyt *et al.*, 2004).

Because of these difficulties, spermatozoa motility alone is not an adequate parameter for predicting boar fertility and extender quality. Indeed, the FMS test demonstrated that the mitochondrial sheath was undamaged in most spermatozoa, confirming the ejaculate quality of the boars used in this study. Spermatozoa with undamaged mitochondria are potentially mobile in spite of any temporary immobility. Therefore, we assumed that reduced rate of motility was temporary.

Our results showed that the velocity values (VCL, VAP and VSL) as well as LIN and STR were high in extenders BTS and Tri-X-Cell which were considered to be providing the best preservation at the end of storage. There was a highly significant correlation between VCL and percentage of sperm motility (r^2^ > 0.9), whilst correlation between VSL and percentage of sperm motility was low (r^2^ < 0.8). The correlation between percentage of motile sperm and measures of sperm movement (ALH, STR, and LIN) was not significant. This suggests that when the percentage of motile cells is elevated the percentage of cells with high velocity is also greater. However, it is more important to know how the spermatozoa are moving because the population is rather variable, containing cells with progressive motility, roundabout motility and with wave motility (Love *et al.*, 2003). The value of AHL is very interesting because this value increases as the velocity decreases and lateral head amplitude and wave motility increases.

After analyses of all motility parameters, the extenders used in this study were ranked according to ability to maintain sperm viability and mitochondrial activity as follows: BTS, MR-A 3, Androstar, Mr Pig Sire, Modena 1, ZM for short-term extenders and Tri-X-Cell, HP2000, Sus for long-term extenders.

Unfortunately, the quantitative composition of these extender media is unknown but we know that boar spermatozoa are very sensitive to the environment, pH, ionic strength, type of ions and osmotic pressure of the medium. One of the extenders used was BTS that is the most widely used semen extender throughout the world. We know that this extender is characterised by containing a small amount of potassium. This feature preserves the sodium potassium pump and thus avoids intracellular potassium depletion which is related to reduced sperm motility (Alvarez and Storey, 1982). Boar spermatozoa can tolerate a fairly wide range of osmotic pressures (240-380 Osm/Kg) but it appears that slightly hypertonic extenders (300 Osm/Kg) provide the best results and extender BTS was slightly hypertonic (325/327 Osm/Kg).

As regards pH, Johnson *et al*. (2000) highlighted that the pH of freshly ejaculated boar semen is around 7.4 ± 0.2, and when this pH is reduced, both metabolism and motility of the spermatozoa are decreased. A similar relationship between pH and motility has been observed in other species (Goltz *et al.*, 1988; Jones and Bavister, 2000). Rodriguez-Martinez *et al.*. 1990 determined that the pH of the caudal part of boar’s epididymis is 6.5 and that mixing spermatozoa with secretions of the accessory glands increases extracellular pH and bicarbonate concentration and this renders spermatozoa fully motile.

The preservation of spermatozoa in the caudal part of the epididymis at lower pH and the observations of a higher motility at higher pH range might explain the decreased motility seen when boar spermatozoa are diluted in a medium with low pH. Under *in vivo* conditions, the increase in pH is a normal, required event during capacitation to induce hyperactivation (Visconti *et al.*, 1998; Schmidt and Kamp, 2004; García Herreros *et al.*, 2005). However, premature induction of these processes will decrease sample quality, and consequently, fertility.

Although differences in pH values were detected in different extenders, it was surprising that these differences did not influence the rate of acrosome reaction. From our analysis, extender BTS has a pH which varies between 6.96 and 6.97. The ideal combination of pH and osmolality could explain its enhanced preservation of boar semen motility compared with others extenders.

In this study, the best long-term extender was Tri-X-Cell with higher osmolality but with average values of pH compared with the other two extenders (pH 6.7.0/6.75 and Osm/Kg 291/296).

Motility was also the parameter used to evaluate the thermoresistance of boar spermatozoa. This test can be considered an accurate clinical test for predicting the fertilizing potential of spermatozoa *in vitro* (Larsson and Einarsson, 1976). In all samples, the reduction in motility was significant and progressive over time. The motility results in extender Tri-X-Cell were more stable, while in extender BTS after 24 hours at 37°C, the percentage of motile cells was 52.56 ± 2.04%.

In every extender, the reduction in motility was evident between the fourth and eighth hours of incubation: this could be explained by higher initial motility due to a rapid uptake of ATP and increased metabolic activity whilst at physiological temperatures. Once again, BTS and Tri-X-Cell appeared most effective at promoting the survival of spermatozoa at physiological temperatures typical of the female genital tract.

The percentage of spermatozoa with no damaged membranes (e.g. intact plasmatic membrane, intact acrosome and high mitochondrial membrane potential) obtained with FMS, was positively correlated with the percentage of motile spermatozoa obtained using CASA. As storage time increased, the percentage of motile spermatozoa decreased in all extenders, and the percentage of nonviable spermatozoa with a low membrane mitochondrial potential and/or spontaneous acrosome reaction increased.

At the end of the storage period a higher percentage of nonviable spermatozoa was recorded in extenders ZM and Sus, whilst BTS and Tri-X-Cell preserved the viability of spermatozoa at the 50% until day 6 and 8 respectively. Our data are in agreement with Hofmo *et al*. (1998) that noted no significant differences related to the use of BTS to preserve semen for 2–3 days compared with another long extender (MR-A) for 4–5 days, and with Waterhouse *et al*. (2004) that demonstrated that the membrane quality of spermatozoa stored in X-cell for 5 days was not significantly different from semen stored in BTS for 3 days. On the contrary, our data are in disagreement with De Ambrogi *et al*. (2006) that detected a decrease of viability in Tri-X-Cell only after 264 hours. The differences between reported data may be attributed to factors other than extenders.

As the duration of storage increased not only did the percentage of non-viable spermatozoa increase and the percentage of vitality spermatozoa decrease, but the percentage of positive cells in the propidium iodide test with a high mitochondrial membrane potential also increased. These cells were not considered to be non-viable, but it was thought that the plasmatic membrane was destabilizing in these cells and that damage to mitochondrial membrane might occur later. The FMS showed how identification of damage simultaneously to different membranes can show subpopulations that not usually recognized by other staining techniques.

The reduction in percentage of destabilized cells in favour of dead cells is shown in Fig. 7 and was similar for all extenders studied. It is important to emphasise that in all extenders studied, there were virtually no live spermatozoa with spontaneous acrosome reactions, and that each extender analyzed could preserve sperm fertilizing capacity for short and long-term storage of fresh semen at 19°C and give good results in artificial insemination *in vivo*. However, if we look at destabilized spermatozoa, it can be seen that this subpopulation is more vulnerable to acrosome reactions (4% and 14% in different extenders). This result, obtained by FMS, would help to identify the subpopulation in fresh semen that is more susceptible to acrosome reaction during preservation and to classify semen in relation to the number of spermatozoa in this category.

In our analysis, the number of CFU remained more or less constant during the study period for all extenders. This phenomenon can be attributed to the presence of antibiotics in the medium.

In conclusion, viability of boar spermatozoa appears to be significantly affected by the kind of extender used and the better extenders such as BTS and Tri-X-Cell can guarantee good preservation of semen viability. This is important since, in the swine industry, semen is preserved both for short and long periods and the choice of extender is important because this may affect the fertilizing capacity of the semen (Dubé *et al.*, 2004).

Moreover, both CASA and FMS techniques gave not only objective information, but also exhaustive information on the characteristics of movement of a single spermatozoon and on the integrity of its membrane. The ability to detect subpopulations by FMS could be useful in classification of fresh ejaculates before preservation and to decide whether to use the ejaculate as a fresh sample or to preserve it in an extender.
